# Thiopurine Exposure During Pregnancy is Not Associated With Anemia in Infants Born to Mothers With IBD

**DOI:** 10.1093/crocol/otad066

**Published:** 2023-10-27

**Authors:** Fiona Yeaman, Amelie Stritzke, Verena Kuret, Nastaran Sharifi, Cynthia H Seow, Amy Metcalfe, Yvette Leung

**Affiliations:** Department of Medicine, University of Calgary, Calgary, AB, Canada; Internal Medicine, University of Western Australia, Perth, WA, Australia; Department of Pediatrics University of Calgary, Calgary, AB, Canada; Department of Obstetrics and Gynecology, University of Calgary, Calgary, AB, Canada; Department of Medicine, University of Calgary, Calgary, AB, Canada; Department of Medicine, University of Calgary, Calgary, AB, Canada; Department of Community Health Sciences; University of Calgary, Calgary, AB, Canada; Department of Medicine, University of Calgary, Calgary, AB, Canada; Department of Obstetrics and Gynecology, University of Calgary, Calgary, AB, Canada; Department of Community Health Sciences; University of Calgary, Calgary, AB, Canada; Department of Medicine and Division of Gastroenterology, University of British Columbia, Vancouver, BC, Canada

**Keywords:** inflammatory bowel disease, thiopurine, pregnancy, neonatal anemia

## Abstract

**Background:**

Thiopurines are commonly used to treat inflammatory bowel disease (IBD). Thiopurines are considered safe throughout pregnancy. However, a published study suggested the risk of neonatal anemia was increased if exposed to thiopurines in utero. This prospective cohort study aimed to determine if there is an increased risk of cytopenia among infants born to pregnant people with IBD, exposed or unexposed to thiopurines, compared to infants born to those without IBD.

**Methods:**

Pregnant IBD patients, with and without thiopurine exposure, and one cohort of control individuals were recruited over a 5-year period. Consenting individuals completed a questionnaire and infants had a complete blood cell count at the newborn heel prick. Anemia was defined as hemoglobin (Hb) < 140g/L. Descriptive statistics were used to characterize the study population. Fisher exact tests were used to examine differences in outcomes between groups, a *P-*value of < 0.05 was deemed significant.

**Results:**

Three cohorts were recruited: 19 IBD patients on thiopurines, 50 IBD patients not on thiopurines, and 37 controls (total of 106). Neonatal median Hb was not different with 177g/L (IQR 38g/L) for the IBD thiopurine group, 180.5g/L (IQR 40g/L) for the IBD non-thiopurine group, and 181g/L (IQR 37g/L) for the controls. Nineteen infants (18%) were cytopenic with 12 (11%) anemic, 6 (5.6%) thrombocytopenic, and 1 (0.94%) lymphopenic. Thiopurine exposure was only in one, mildly anemic, infant.

**Conclusions:**

These findings further support physicians and IBD patients contemplating pregnancy that current guidelines recommending thiopurine adherence do not lead to increased perinatal risk of anemia or cytopenia.

## Introduction

Thiopurines (azathioprine, 6-mercaptopurine, thioguanine) are a class of immunomodulator medications commonly used in the treatment of inflammatory bowel disease (IBD). As IBD is often diagnosed during reproductive years, thiopurines are often used in pregnant people.^1,2^ Disease activity in pregnancy is the strongest predictor of adverse outcomes^3^; therefore current pregnancy IBD guidelines recommend patients continue with thiopurine therapy during pregnancy to maintain disease stability and avoid flares.^4^

Multiple studies dating back more than three decades have shown thiopurines to have no consistent negative effect on IBD pregnancies in terms of congenital anomalies or neonatal morbidity.^5–10^ Additionally, in a prospective IBD cohort of 108 pregnancies there was no association between thiopurine use and adverse birth or health outcomes in infants up to one year of age.^11^ Longer-term follow-up studies have also found no adverse effect on physical and psychosocial development in children.^12^

However, a 2014 study of sixteen pregnancies reported that 63% of infants (10/16) born to parents exposed to thiopurines were deemed anemic at birth with a median hemoglobin (Hb) concentration of 9.25 mmol/L (149g/L), IQR 8.25–9.60 mmol/L (133–155g/L).^13^ Concerned physicians may be more inclined to measure hemoglobin in infants exposed to thiopurine in utero, or even consider the discontinuation of thiopurine therapy in pregnant people with IBD. This is problematic as there is a substantial risk of disease flare when thiopurines are discontinued during pregnancy, with relapse rates following discontinuation of thiopurines ranging from 14% to 59% in the first year.^14–16^ The cited study was small (16 pregnancies) and, it did not include a non-thiopurine control group. It is unknown whether it may be the disease itself or the thiopurine exposure that may be associated with newborn anemia. Furthermore, the authors did not provide data on the timing of when the hemoglobin level was drawn post-delivery or the timing of umbilical cord clamping, two factors that directly impact the normal expected range of hemoglobin.^17^

Our aim was to conduct a prospective observational cohort study to determine whether there is an increased risk of anemia, or other cytopenias, among infants born to people with IBD exposed or unexposed to thiopurines, compared to infants born to people without IBD.

## Methods

Pregnant people with IBD were recruited from the IBD and Pregnancy Clinic from April 2013 to July 2018. This clinic prospectively enrolls women with IBD and follows them during the preconception, pregnancy, and postpartum periods. The physicians in this clinic use a standardized clinical care pathway to ensure consistent patient education about the safety of medications and to closely monitor their disease with the goal of steroid-free clinical remission during this critical time period. Disease activity is assessed by validated disease activity indices, the Harvey Bradshaw Index (Crohn’s disease) and the Simple Clinical Colitis Activity Index (ulcerative colitis or indeterminate colitis); IBD medications are recorded at each visit; and pregnancy complications and pregnancy outcomes are captured using standardized questionnaires.

Two cohorts of IBD patients were enrolled; one with exposure to thiopurines, and the other without exposure to thiopurines. Non-thiopurine IBD patients may have had exposure to biologic IBD treatments including anti-TNF, vedolizumab, and ustekinumab.

A third cohort of controls without IBD, with pregnancies receiving obstetrical care from the adjacent pregnancy clinic at the same center, were recruited between 2015 and 2018 and prospectively followed until delivery.

Pregnant individuals were not eligible to participate in this study if there was a history of anemia in the mother within 3 months of conception, acute perinatal blood loss, known congenital or chromosomal fetal anomaly, or hemolysis of the newborn due to alloimmune antibodies.

Upon recruitment, individuals were asked to complete a brief questionnaire and consent to a complete blood cell count (CBC) for their infants to be done at the time of the newborn heel prick for the Newborn Metabolic Screen, a universal screening program for all infants within 24–72 hours after birth. A neonatal specialist who did not have access to the in utero medication exposure then separately reviewed each case of cytopenia to determine the likely cause of abnormality.

We defined cytopenias as the following, based on local laboratory figures: hemoglobin < 140g/L for anemia, white blood cell count < 1.5 × 10^9^/L for neutropenia, and platelet count < 100 000 × 10^9^/L for thrombocytopenia.

The prevalence of neonatal anemia is approximately 2%–3% adjusting for cord clamping at our tertiary center. To detect a clinically meaningful, increased rate of 20% for anemia in infants exposed to thiopurines, we calculated that we would need a sample size of 38 exposed and 38 unexposed pregnancies. To add strength to the comparison, two different unexposed cohorts were sought, one with IBD and one without IBD. Descriptive statistics were used to characterize the study population. Fisher exact tests were used to examine differences in outcomes between groups, a *P-*value of <.05 was deemed significant. Ethics approval for this study was obtained from the institutional review board.

## Results

Overall, 106 adult individuals were recruited into this study and maintained enrollment until the time of the newborn heel prick testing.

These 106 individuals included 69 patients with IBD, and 37 in the control group without IBD and with no exposure to thiopurines nor other immunomodulators for any other indication. Of the 69 individuals with IBD, 19 were exposed to thiopurines during their pregnancy, and 50 individuals with IBD were not exposed. One pregnancy was multiparous with twins being born in the IBD without thiopurine exposure cohort. Population baseline characteristics are described in Table 1. There were no significant differences in baseline characteristics between the IBD and control groups, except there was a statistically significant higher proportion of multiparous pregnancies in the control group (42.1% IBD thiopurine; vs 46.0% IBD non-thiopurine; 89.2% control *P* < .001).

**Table 1.** Participant demographics.

Parameter	IBD—thiopurine *N* = 19 *N* (%, 95% CI)	IBD – no thiopurine *N* = 50 *N* (%, 95% CI)	Control *N* = 37 *N* (%, 95% CI)	*P*-value
Annual household income ≥$100 000	11 (68.8, 43.0–86.5)	21 (61.8, 44.5–76.5)	21 (58.3, 41.7–73.3)	.775
Completed an undergraduate degree	16 (84.2, 60.6–94.9)	34 (68.0, 53.8–79.5)	21 (56.8, 40.5–71.7)	.115
Caucasian	13 (86.7, 59.0–96.7)	30 (85.7, 69.7–94.0)	29 (82.9, 66.5–92.2)	.921
Maternal age ≥ 35	5 (26.3, 11.3–50.0)	17 (34.0, 22.2–48.2)	9 (24.3, 13.1–40.7)	.589
Multiparous	8 (42.1, 22.5–64.6)	23 (46.0, 32.7–59.9)	33 (89.2, 74.3–95.9)	<.001

Table 2 describes the two IBD populations: There were no differences in disease activity in the third trimester of pregnancy, nor in the proportions of patients with IBD-related surgery and perianal disease between the IBD thiopurine and IBD non-thiopurine groups. Within the 19 IBD pregnancies with thiopurine exposure 6/19 (31.6%), were on combination therapy with infliximab or adalimumab. 27/50 (54%) of the non-thiopurine IBD patients were on biologic monotherapy (infliximab, adalimumab, golimumab, ustekinumab, or vedolizumab). In a minority of patients, some data were not readily available from chart review.

**Table 2.** IBD characteristics.

	IBD—thiopurine *N* = 19 *N* (%, 95% CI)	IBD—no thiopurine *N* = 50 *N* (%, 95% CI)	*P*-value
Crohn’s disease distribution			.096
Colonic	4 (33.3, 12.7–63.3)	8 (28.6, 14.6–48.2)	
Ileal	6 (50.0, 23.7–76.3)	6 (21.4, 9.7–40.9)	
Ileo-colonic	2 (16.7, 4.0–48.9)	14 (50.0, 31.8–68.2)	
Crohn’s disease behavior			.143
Fibrostenotic	5 (41.7, 17.9–70.0)	5 (19.2, 8.0–39.5)	
Inflammatory	7 (58.3, 30.0–82.1)	16 (61.5, 41.4–78.4)	
Penetrating	0	5 (19.2, 8.0–39.4)	
Perianal^*^	5 (45.5, 19.6–74.0)	4 (16.0, 5.9–36.6)	.060
Ulcerative colitis			.178
Left-sided	1 (20.0, 2.4–71.8)	6 (35.3, 16.0–61.0)	
Pan-colonic	4 (80.0, 28.2–97.6)	6 (35.3, 16.0–61.0)	
Proctitis	0	5 (29.4, 12.1–55.8)	
History of IBD-related surgery	3 (17.6, 5.7–43.3)	11 (23.9, 13.6–38.5)	.595
Has a pouch	0	3 (20.0, 6.0–49.4)	.396
History of extraintestinal manifestations	5 (31.3, 13.4–57.3)	15 (39.5, 25.1–55.9)	.568
Taking a biologic medication	6 (31.6, 14.7–55.3)	27 (54.0, 40.0–67.4)	.096
Anti-TNF	6	25	
Vedolizumab	0	1	
Ustekinumab	0	1	
Taking 5-ASA	7 (36.8 (18.4–60.1)	20 (40.8, 27.9–55.2)	.764
Disease activity			.822
Active	2 (10.6, 2.6–34.4)	6 (12.5, 5.6–25.5)	
Remission	17 (89.5, 65.6–97.4)	42 (87.5, 74.5–94.4)	

^*^Inactive for all.

There was no difference in birth outcomes in terms of preterm delivery (<37 weeks of gestation) for the offspring. In the IBD thiopurine group, 3/19 (15.8%, 95% CI: 5.1–39.6) were delivered preterm, compared to 11/50 (22.0%, 95% CI: 12.4–35.7) in the IBD non-thiopurine group, and 4/37 (10.8%, 95% CI: 7.2–41.6), in the control group, *P* = .841.

Similar proportions of pregnancies were delivered by cesarean section: In the IBD thiopurine group 11/19 (57.9%, 95% CI: 40.1–83.4); in the IBD non-thiopurine group 23/50 (46.9%, 95% CI: 33.4–61.0); and in the control group 16/37, (44.4%, 95% CI: 29.1–60.9), *P* = .357.

There was no difference in need for admission to the neonatal intensive care unit: The IBD thiopurine group had 2/19 (10.5%, 95% CI: 3.5–43.2) admissions; the IBD non-thiopurine group had 10/50 (20.0%, 95% CI: 12.6–37.5); and the control group had 8/37 (21.6%, 95% CI: 11.1–37.9), *P* = .792.

The majority of infants (92%) in all three groups had no severe abnormalities detected in their CBCs (normal, or mild anemia only). Table 3 summarizes the neonatal cytopenia outcomes with their CBC results. There were no differences between the groups regarding occurrence of anemia, leukopenia, or thrombocytopenia. The median Hb for the three groups were 177g/L (IQR 38g/L) for the IBD thiopurine group, 180.5g/L (IQR 40g/L) for the IBD non-thiopurine group, and 181g/L (IQR 37g/L) for the control group. There were 11 cases of mild neonatal anemia (10%), 1 in the IBD thiopurine, 5 in the IBD non-thiopurine, and 5 in the control group (*P* = .725, Table 3). One anemia was deemed severe (Hb < 100g/L) in the IBD non-thiopurine group, and 5 in the control group had severe anemias. Six thrombocytopenia cases (5.7%) and one leukopenia (0.94%) case were identified. The leukopenia was a neutropenia from the IBD non-thiopurine group. Three thrombocytopenia infants were from the IBD non-thiopurine group and three were from controls. No infant had more than one cytopenia and none of the cytopenia cases required intervention.

**Table 3.** Neonatal CBC results by thiopurine exposure.

	IBD—thiopurine *N* = 19 *N* (%, 95% CI)	IBD – no thiopurine *N* = 50 *N* (%, 95% CI)	Control *N* = 37 *N* (%, 95% CI)	*P*-value
No abnormalities were detected via CBC	18 (94.7, 70.1–99.3)	40 (80.0, 66.5–89.0)	29 (78.4, 62.2–88.9)	.297
No severe abnormalities were detected via CBC	19 (100)	45 (90.0, 77.9–95.8)	34 (91.9, 77.4–97.4)	.543
Neonatal Anemia (hemoglobin < 140 g/l)	1 (5.2, 0.7–29.9)	5 (12.0, 5.4–24.4)	5 (13.5, 5.7–28.8)	.725
Severe Neonatal Anemia (hemoglobin < 100 g/l)	0	1 (2.0, 0.3–13.1)	0	1.00
Polycythemia (hct > 0.7)	0	0	0	1.00
Thrombocytopenia (platelets < 100 × 10^9^/L)	0	3 (6.0, 1.9–17.2)	3 (8.1, 2.6–22.6)	.564
Neutropenia (neutrophils < 1.5 × 10^9^/L)	0	1 (2.0, 0.3–13.1)	0	1.00
Cytopenia requiring specific treatment	0	0	0	1.00

Table 4 outlines the 11 cases of cytopenias in offspring of IBD parents found with available information from the hospital charts. Only 1 of the 11 cytopenias in infants of IBD patients was exposed to thiopurines in utero, and they were also exposed to biologic medication (adalimumab) and had a hemoglobin of 139g/L. This infant was born at term via elective cesarean section due to previous maternal fourth^-^degree tear, and had Apgars of 9 and 9. Half of the cases of anemia detected were thought to be secondary to a suspected infection, determined by neonatologist assessment.

**Table 4.** Cytopenia cases in infants of IBD pregnancies.

IBD type	Cytopenia	Gestational age (weeks)	Birthweight (grams)	Thiopurine	Biologic	Associated conditions
CD	Anemia (120 g/L)	40	2560	No	Anti-TNF (golimumab)	Influenza
CD	Anemia (122 g/L)	36 + 5 days	2930	No	No	Suspected sepsis
CD	Anemia (139 g/L)	38 + 5 days	Appropriately grown	Yes	Anti-TNF (adalimumab)	None
CD	Anemia (135 g/L)	40 + 1 day	3285	No	Anti-TNF (infliximab)	Subgaleal hemorrhage
UC	Anemia (135 g/L)	41 + 1 day	4023	No	Anti-TNF (infliximab)	Suspected sepsis
UC	Anemia (119 g/L)	40	3390	No	Anti-TNF (infliximab)	Perinatal brain injury
UC	Anemia (severe) (87 g/L)	35 + 3 days	3066	No	No	Rh incompatibility
CD	Thrombocytopenia (48 × 10^9^/L)	40	3106	No	Anti-TNF (adalimumab)	Normal when rechecked
UC	Thrombocytopenia (21 × 10^9^/L)	37	2680	No	Anti-TNF (infliximab)	Clumped platelets
UC	Thrombocytopenia (74 × 10^9^/L)	38 + 5 days	3090	No	No	None
CD	Neutropenia (1.0 × 10^9^/L)	30	1006	No	Anti-TNF (adalimumab)	Twin pregnancy

## Discussion

In our study, we found that there were no differences in proportions of anemia and other cytopenias in infants exposed in utero to thiopurines during IBD pregnancies compared to both IBD pregnancies without thiopurine exposure and control pregnancies.

Thiopurines have been a mainstay of maintenance treatment in IBD since the 1990s, having first been published in 1974.^14,18,19^ More recently, in the biologic era, thiopurine monotherapy remains an effective and cost-efficient maintenance treatment for IBD, particularly in moderate ulcerative colitis.^20,21^ Thiopurines can also be used in combination with anti-TNF agents for moderate to severe IBD.^22^ The presumptive active 6-thioguanine nucleotides (6-TGN) inhibit purine nucleotide metabolism and therefore are potentially myelotoxic.^23,24^ Thiopurine metabolites are transferred across the placenta^25^ and 6-TGN is detectable in the red blood cells of infants exposed in utero.^13,26^ Alterations in thiopurine metabolite levels by trimester have been observed; with 6-TGN levels being lower and 6-MMP being higher during pregnancy compared to preconception.^13,27,28^ Iron accumulation and transplacental supply to the fetus; however, is strictly regulated to maintain normal hemoglobin values even in severe maternal anemia which might prove protective.^29^ Hemoglobin levels of the infants in this study were consistent with reported mean neonatal hemoglobins for term infants.^30^

This study identified 11 cases of cytopenias in the offspring of IBD mothers; only one of which was exposed to thiopurines. The infant with thiopurine exposure and an anemia also had exposure to adalimumab. This infant had very mild anemia (139g/L) and was born at term without complication. Seven of the infants with cytopenias were exposed to anti-TNF agents, including the one exposed to thiopurine. In total, 33 IBD patients were exposed to biologic treatments; 6 in combination with thiopurines and 27 in the non-thiopurine group. There is no evidence to date that biologic treatment is contributory to neonatal cytopenias.^31,32^ Transplacental transfer of anti-TNF (infliximab, adalimumab) increases throughout pregnancy as IgG monoclonal antibodies are able to cross the placenta as it matures^33^; but this has not transcribed to an increase in infection risk in offspring.^34^

Several babies had cytopenias attributed to infections, all with anemias rather than leukopenias. This would make the anemia more likely reactive than as a consequence to in utero therapies. Anemia is a common finding in neonatal infection.^35^ Only one baby had a neutropenia; this was in the setting of twin pregnancy and this baby was not exposed to thiopurines. There has been one case reported of severe lymphopenia associated with a thiopurine-exposed IBD pregnancy.^36^ A study of 11 infants exposed to thiopurines due to maternal disease including IBD, lupus, renal transplant, vasculitis, and interstitial lung disease found when adjusted for gestational age, normal hemoglobin concentrations and only one case of neutropenia, which was attributed to chorioamnionitis.^37^ A study of older infants between nine and 15 months old born to mothers with IBD showed no differences in rates of anemia at this later stage of infancy in 21 infants exposed in utero to thiopurines compared to 13 not exposed in utero.^38^ A prospective study of 20 maternal and fetal thiopurine levels and outcomes identified one neonatal hemoglobin of 128g/L that resolved by 6 weeks of age, and no other anemias.^28^ In a more recent Dutch study looking at tioguanine in pregnancy, 16 of the 117 babies had blood counts taken at birth demonstrating two anemias (13%) and no infants with leukopenia or thrombocytopenias.^39^

The Jharap study used a limit of less than 10 mmol/L (161g/L) to define neonatal anemia.^13^ This is higher than the reference range at our center with a cutoff of < 140g/L. This higher cutoff will have increased their anemia rates, possibly falsely, particularly when severe anemia (Hb < 100 g/L) is clinically relevant. Only one infant in our cohort had a severe anemia, which was macrocytic with MCV 147 fL. They were not exposed to thiopurines, nor any IBD medication. The mother had a pouch following previous ulcerative colitis and her only medication was fluoxetine.

This study has both strengths and limitations. Strengths of our study are the inclusion of an IBD group with no exposure to thiopurines and a non-IBD control group. To date, this is the only study to the best of our knowledge to have included a non-IBD control group to compare outcomes with and it is the largest series of neonates born to IBD parents with neonatal thiopurine exposures to date.^13,28,39^ Furthermore, we included an IBD non-thiopurine group with similar disease activity, and disease complications to tease out disease versus therapy effect. Offspring from this non-thiopurine IBD group more commonly exhibited cytopenias than those in the thiopurine-exposed group. It may be that previous findings of cytopenias were secondary to the mothers’ chronic systemic disease of IBD rather than secondary to exposure to thiopurines and other medications.

There were no differences in newborn outcomes such as preterm birth and admission to neonatal intensive care unit between all three groups; therefore controlling for the possible confounder of neonatal illness causing cytopenia. To date, previous studies have not controlled for timing of cord clamping which has a direct effect on the interpretation of newborn red cell blood counts in contrast to our study where >95% in our health region clamp between 30 to 60 seconds after delivery. The difference in neonatal hematocrit, to account for in the setting of delayed cord clamping, is expected to be around 6% higher if cord clamping is delayed compared to immediate.^17^ Additionally, all our blood samples were taken as part of the universal newborn metabolic screen therefore limiting bias that bloodwork was only done for clinically suspicious cases. The addition of a CBC was done as standard of care to address the possibility of cytopenias.

Conversely, despite the long study period, we were not able to meet our calculated power of 38 in each group. The lower proportion of non-IBD pregnancies occurred due to the difficulty of finding participants who were enrolling for completely altruistic reasons which delayed the data analysis. The thiopurine group was also smaller than intended as there was a larger proportion of IBD pregnancies not exposed to thiopurines compared to IBD pregnancies exposed to thiopurine due to the general population of our IBD pregnancy clinic within a tertiary center. With a background prevalence of neonatal anemia of approximately 1.5%, 80 pregnancies would be needed to detect a rate of neonatal anemia equivalent to that reported in the Jharap study; therefore, it is possible that our study is underpowered. Although smaller than planned, we still report prospective, controlled data showing no significant difference between thiopurine-exposed and unexposed infants with regard to anemia and cytopenia in general which is encouraging.

Chart review showed whether patients were on thiopurines or not each trimester, but we did not monitor for adherence to medications in this study with metabolite levels. The thiopurine cohort were patients of an IBD-specific pregnancy clinic where appropriate counseling is carried out which has been shown to improve adherence. There is significant evidence to support the safety of thiopurines in pregnancy and guidelines recommending continuation,^4^ but despite this, adherence to thiopurines during pregnancy has been found to be less than biologic therapies.^40^

Metabolites were not checked in this study as metabolites require more complex testing that is outside standard of care and it may be possible to have anemia with normal metabolites.^41^

## Conclusion

In conclusion, thiopurine exposure was only present in one cytopenic baby (mild anemia) of all cytopenias identified. These negative findings regarding cytopenias in neonates with thiopurine exposure in utero will help to reassure both physicians and patients contemplating pregnancy with IBD that current guidelines recommending thiopurine adherence do not lead to increased perinatal risk. This will hopefully avoid unnecessary cessation of an important therapy maintain remission during pregnancy and mitigate the consequent risks of disease flare on both mother and baby. In keeping with current guidelines, we recommend that for IBD patients on thiopurine during pregnancy, patients stay on their prescribed thiopurine therapy to avoid disease flare during pregnancy. For offspring of pregnancies complicated by IBD, there is no evidence to suggest routine CBCs are warranted; instead, they should be utilized only when clinically indicated. None of our participants required intervention or therapy for exposure to thiopurines. Increased surveillance and prudent treatment in the setting of preterm birth and other neonatal illnesses is warranted in infants of IBD pregnancies, as in all pregnancies.

## Data Availability

De-identified data are available upon reasonable request to the corresponding author (FY).
